# Extracellular enzyme activity of entomopathogenic fungi, *Beauveria bassiana* and *Metarhizium anisopliae* and their pathogenicity potential as a bio-control agent against whitefly pests, *Bemisia tabaci* and *Trialeurodes vaporariorum* (Hemiptera: Aleyrodidae)

**DOI:** 10.1186/s13104-022-06004-4

**Published:** 2022-03-26

**Authors:** Amha Gebremariam, Yonas Chekol, Fassil Assefa

**Affiliations:** Department of Microbial, Cellular, and Molecular Biology, College of Natural and Computational Science, Addis Ababa University, Addis Ababa, Ethiopia

**Keywords:** Biological control, Enzyme activity index, And mortality

## Abstract

**Objective:**

This study was aimed to assess the enzymatic activity and pathogenicity potential of *Beauveria bassiana* and *Metarhizium anisopliae* against whiteflies in Ethiopia.

**Results:**

The data showed that *Beauveria bassiana* AAUMB-29, AAUMFB-77, and AAUEB-59 generated the highest chitinase (EI = 3.41), lipase (EI = 4.45), and protease activities (EI = 5.44) respectively. The pathogenicity study of isolates on whitefly nymphs and adults indicated significant variation (P < 0.05) with mortality ranging from 71.67 to 98.33% and 60 to 100% against *Bemisia tabaci* and *Trialeurodes vaporariorum* nymphs respectively. The mortality of adults was between 58 and 94.27% against *B. tabaci* and 59.03 to 95.37% against *T.vaporariorum.* The result also showed that AAUMB-29, AAUMFB-77, and AAUDM-43 were the most virulent with LC_50_ values of 2.7 × 10^4^, 5.3 × 10^4^, and 5.4 × 10^4^ conidia/ml against nymphs of *B. tabaci*, and with LC_50_ values 6.8 × 10^4^, 8.2 × 10^4^, and 7.2 × 10^4^ conidia/ml against nymphs of *T. vaporariorum*, respectively. The *B. bassiana* AAUMB-29, *B. bassiana* AAUMFB-77, and *M. anisopliae* AAUDM-43 induced the highest whitefly mortality than other isolates. These isolates can be recommended for further tests under field conditions to fully realize their potential as effective biocontrol agents against whitefly pests in tomato.

**Supplementary Information:**

The online version contains supplementary material available at 10.1186/s13104-022-06004-4.

## Introduction

Whiteflies *Bemisia tabaci* and *Trialeurodes vaporariorum* (Hemiptera: Aleyrodidae) are notorious sap-sucking pests causing serious damage to vegetable crops through direct feeding and transmission of several plant viruses [1]. Tomato (*Solanum lycopersicum*) is one of the most favorable hosts of *B. tabaci* and *T. vaporariorum* [2], and these whitefly species cause tomato yield losses from 50 to 100% [3]. The control of whitefly is mainly dependent upon chemical pesticides to reduce agronomic losses [4, 5]. However, the use of chemical pesticides induces pest resistance and an outbreak of secondary pathogens [6].

Entomopathogenic fungi (EPF) *Beauveria bassiana* and *Metarhizium anisopliae* are the most environmentally-friendly bio-control agents against sucking insect pests [7]. They produce adhesion factors, cuticle degrading enzymes, infection structures [8], and toxic secondary metabolites to overcome host cuticles and cause infection [9]. These typical features of *B. bassiana* and *M. anisopliae* provide an advantage to effectively control sap-sucking insect species over others [10]. Different laboratory and field studies demonstrated that isolates of *B. bassiana* and *M. anisopliae* effectively controlled *B. tabaci* and *T. vaporariorum* with mortality ranging from 71 to 96.61% [11–13].

Increased interests in the use of entomopathogenic fungi in pest management options necessitate the selection of fungal isolates with high virulence that shows significant enzyme activities on target insects. In Ethiopia, locally isolated entomopathogenic fungi showed promising results for the control of agricultural pests such as *Aphis gossypiin* [14], *Pachnoda interrupta* [15], and *Tuta absoluta* [16]. However, there is a limited report on the use of these native isolates for the management of whiteflies. Hence, this study was carried out to evaluate the enzymatic activity and pathogenicity competence of *B. bassiana* and *M. anisopliae* against whitefly species, *B. tabaci* and *T. vaporariorum* in Ethiopia.

## Main text

### Material and methods

Indigenous entomopathogenic fungi *B. bassiana* and *M. anisopliae* were used in trials (Additional file 1: Table S1). Isolates were obtained from soil samples collected from farmlands and forest sites of Ethiopia. The potential isolates were selected based on their virulence effectiveness [17].

### Cuticle degrading enzyme production with agar plate methods

The 5 mm mycelial agar disc of each isolate was transferred in triplicates into casein hydrolysis agar composed of; (KH_2_PO_4_ (1 g), KCl (0.5 g), MgSO_4_·7H_2_O (0.4 g), CaCl_2_·2H_2_O (0.1 g), powdered skim milk (25 ml of 15%), glucose (10 g), agar (12 g) and distilled H_2_O (1000 ml) [18], and incubated at 25 °C for 10 days to evaluate their protease activity.

Isolates were screened for chitinase activity on the chitin-agar medium according to the method suggested by Maketon et al. [19]. The 5-mm mycelial agar disc of each isolate was transferred in triplicates to the chitin-agar medium composed of; (NH_4_)_2_ SO_4_ (1 g), K_2_HPO_4_ (1 g), KCl (0.5 g), NaCl (5 g), and MgSO_4_ (0.5 g), FeSO_4_ (0.01 g), agar–agar (20 g), colloidal chitin (5 g) and distilled H_2_O (1000 ml). They were incubated at 25 °C for 10 days.

Isolates were screened for lipase activity according to Falony et al*.* [20]. The 5 mm mycelia gar discs were inoculated into a basal medium in triplicates with a composition (g/L): NaH_2_PO_4_ 1.2, MgSO_4_·7H_2_O 0.3, KH_2_PO_4_ 2, CaCl_2_ 0.25, 0.003% NaCl, 2% agar, (NH_4_)_2_SO_4_ at 1%, and olive oil at 2% and incubated at 25 °C for 10 days. Enzymatic index (EI) was calculated using the following formula [21]:

$$ {\text{Enzymatic Index }}\left( {{\text{EI}}} \right) = \frac{{\text{Hydrolysis zone diameter}}}{{\text{Colony growth diameter}}}. $$

### Pathogenicity test against whitefly nymphs and adults

Adult and nymph whiteflies were released to tomato leaves containing sprayed residues of fungal isolates as described by Mascarin et al. [22]. Tomato leaves were sprayed with 3 ml of a conidial suspension of isolates at 1 × 10^7^ conidia/ml. After spraying, leaves were placed onto 0.2% water agar in a petri dish and adult whiteflies were released into treated leaves (15 adults/leaf) in triplicates and incubated at 25 °C for 10 days. Similarly, the mortality of whitefly nymphs was assessed by spraying leaf discs (30 mm in diameter) containing 20 nymphs with 3 ml of conidial suspension of 1 × 10^7^ conidia/ml. Then leaf discs were placed onto 0.2% water agar medium in a Petri dish and incubated at 25 °C for 10 days. The median of lethal time (LT_50_) of each isolates at 10 days of post inoculation was calculated using probit analysis.

### Sporulation of isolates on whitefly nymph cadavers

The spore production of isolates was assessed according to Mascarin et al. [22]. To quantify yield, four sporulated nymphs were randomly selected within each treatment and transferred into a 1.8 ml microcentrifuge tube containing 1.5 ml of 0.1% Triton X-100 and from which 1 ml was counted in triplicates using a hemocytometer.

### Multiple-dose responses studies

The multiple-dose bioassay (1 × 10^5^–1 × 10^8^ conidia/ml) was evaluated to estimate the average lethal concentration (LC_50_) values of each isolate [23]. Each treatment was undertaken in triplicates to record nymph mortality for 10 days with periodic observation every day.

### Data analysis

Mortality data were corrected using Abbott’s formula [24]. The corrected mortality and spore per whitefly nymph cadaver were arcsine transformed [25] and subjected to the ANOVA procedure in SPSS version 20. The bioassay evaluation was tested by means separated using Tukey’s HSD test at P < 0.05. The lethal time **(**LT_50_) and the lethal concentration (LC_50_ and LC_90_) values were determined with probit analysis (IBM SPSS statics 20) [26].

## Results

### Cuticle degrading enzymatic activities

Entomopathogenic fungi were showed significant differences in their relative enzyme activities ranging from 1.20 to 3.41 for chitinase, 1.58 to 4.45 for lipase, and 1.72 to 5.44 for protease (Table 1). On average, the isolates displayed the highest protease index (3.41), followed by the lipase index (2.86) and chitinase index (2.42), respectively. Isolates showed significant differences in their enzyme activities; where almost all isolates (92%) showed excellent protease activities while 75% and 50% of the isolates displayed excellent lipase and chitinase activities respectively (Additional file 1: Fig. S1). Although 67% of the isolates showed excellent overall activities, *B. bassiana* AAUMB-29 performed best in chitinase activity (EI = 3.41), whereas *B. bassiana* AAUMFB-77 and *B. bassiana* AAUEB-59 exhibited the highest lipase (EI = 4.45), and protease activity (EI = 5.44) respectively.

**Table 1:** The enzymatic indices of *B. bassiana* and *M. anisopliae* isolates

Isolate code	Species	Enzymatic index						Average index	AREA
		Chitinase index	REA	Lipase index	REA	Protease index	REA		
AAUMFB-5	*B. bassiana*	2.29	G	1.58	F	2.16	E	2.01	G
AAUMFB-77	*B. bassiana*	2.94	E	4.45	E	4.11	E	3.83	E
AAUMB-29	*B. bassiana*	3.41	E	4.10	E	3.11	E	3.54	E
AAUMB-20	*B. bassiana*	2.73	E	2.11	F	2.52	E	2.45	G
AAUEB-59	*B. bassiana*	2.49	G	2.86	E	5.44	E	3.59	E
AAUMB-21	*B. bassiana*	2.29	G	2.80	E	3.63	E	2.91	E
AAUKB-11	*B. bassiana*	2.84	E	3.30	E	3.03	E	3.05	E
AAUDM-43	*M. anisopliae*	2.82	E	3.00	E	2.81	E	2.87	E
AAUZM-60	*M. anisopliae*	1.20	P	1.92	F	2.83	E	1.98	F
AAUEM-30	*M. anisopliae*	1.26	P	1.64	F	1.72	F	1.54	F
AAUMFM-6	*M. anisopliae*	2.17	G	2.60	E	4.49	E	3.08	E
AAUZM-18	*M. anisopliae*	2.70	E	3.99	E	5.08	E	3.92	E
Average		2.42	50%	2.86	75%	3.41	92%	2.90	67%

*REA* relative enzymatic activity, *AREA* average relative enzymatic activity, *E* excellent activity, *G* good activity, *F* fair activity, *P* poor activity

### Virulence of *B. bassiana* and *M. anisopliae* isolates against whitefly adults

**Fig. 1:**
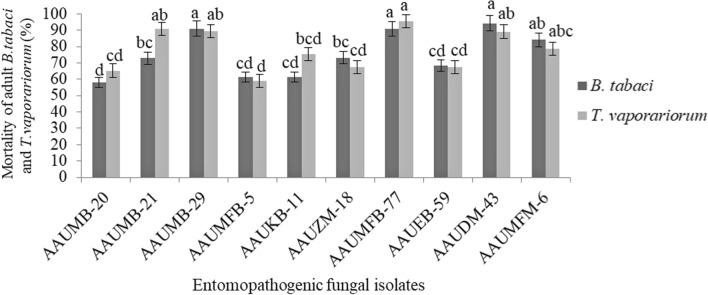
Percent mortality (Mean ± SE) of *B. tabaci* and *T. vaporariorum* adults after 10 days of treatment with *M. anisopliae* and *B. bassiana* at 1 × 10^7^ conidia/ml

All isolates were pathogenic to whiteflies, *B. tabaci*, and *T. vaporariorum* adults (Fig. 1)*.* The different fungal isolates showed mortality of whitefly adults between 58 to 94.27% for *B. tabaci* and 59.03 to 95.37% for *T. vaporariorum* after 10 days treatment. The result showed that *M. anisopliae* AAUDM-43 and *B. bassiana* AAUMFB-77 displayed the highest mortality of 94.27% and 95.37% on *B. tabaci* and *T. vaporariorum* adults, respectively (Additional file 1: Figs. S2, S3).

### Mortality (%), median lethal time (LT_50_), and spore production perspective of isolates on cadavers of whitefly nymphs

The bio-insecticide efficacies of entomopathogenic fungi showed significant differences (P < 0.001) in percentage mortality of *B. tabaci* and *T. vaporariorum* nymphs (Table 2). The mortality of *B. tabaci* and *T. vaporariorum* nymphs varied from 71.67 to 98.33% and 60 to 100%, respectively. Thus, *B. bassiana* AAUMB-29 displayed 98.33% mortality on *B. tabaci*. Similarly, *B*. *bassiana* AAUMB-29 and AAUMFB-77 caused 100% mortality on *T. vaporariorum* nymphs.

**Table 2:** Mortality (%), median lethal time (LT_50_), and spore production per *B. tabaci* and *T. vaporariorum* nymphs after 10 days of treatment with *M. anisopliae* and *B. bassiana* at 1 × 10^7^ conidia/ml

Isolates	% Mortality of *B. tabaci* (Mean ± SE)	LT_50_ (days) (95% CL)	Spore per *B. tabaci* (conidia/ml)	% Mortality of *T. vaporariorum* (Mean ± SE)	LT_50_ (days) (95% CL)	Spore per *T. vaporariorum* (conidia/ml)
AAUMB-20	76.67 ± 1.66^cd^	9.26 (7.94–11.98)	1.3 × 10^5^ ± 4.3 × 10^3d^	81.67 ± 1.66^a^	7.52 (6.51–9.15)	1.6 × 10^5^ ± 8.5 × 10^3d^
AAUMB-21	80.00 ± 2.88^bcd^	3.52 (2.68–4.35)	2.5 × 10^6^ ± 2.4 × 10^5bc^	85.00 ± 2.88^a^	4.35 (3.66–5.03)	3.6 × 10^6^ ± 6.4 × 10^5b^
AAUMB-29	98.33 ± 1.66^a^	3.36 (2.49–4.19)	3.0 × 10^6^ ± 1.3 × 10^5b^	100.00^a^	3.82 (3.17–4.43)	3.7 × 10^6^ ± 2.1 × 10^5b^
AAUMFB-5	80.00 ± 5.77^bcd^	6.24 (5.34–7.10)	3.4 × 10^4^ ± 2.1 × 10^3d^	60.00 ± 5.77^b^	8.16 (6.93–10.48)	2.4 × 10^5^ ± 6.4 × 10^3d^
AAUKB-11	85.00 ± 5.00^abcd^	7.32 (6.72–8.08)	5.6 × 10^4^ ± 2.3 × 10^3d^	86.67 ± 4.40^a^	6.54 (5.83–7.44)	2.0 × 10^5^ ± 2.1 × 10^4d^
AAUZM-18	93.33 ± 1.66^ab^	8.04 (7.38–8.97)	4.7 × 10^5^ ± 6.4 × 10^3d^	81.67 ± 4.40^a^	7.63 (6.77–9.36)	4.4 × 10^5^ ± 3.6 × 10^4d^
AAUMFB-77	91.67 ± 1.66b^abc^	3.22 (2.47–3.93)	6.5 × 10^6^ ± 2.6 × 10^5a^	100.00^a^	3.08 (2.61–3.54)	5.8 × 10^6^ ± 4.1 × 10^5a^
AAUEB-59	71.67 ± 1.66^d^	5.84 (4.82–7.27)	2.2 × 10^5^ ± 1.5 × 10^4d^	88.33 ± 3.33^a^	8.13 (7.55–6.99)	1.9 × 10^5^ ± 2.6 × 10^4d^
AAUDM-43	95.00 ± 2.88^ab^	3.73 (3.09–4.36)	2.0 × 10^6^ ± 1.5 × 10^5c^	95.00 ± 2.88^a^	4.91 (4.39–5.42)	1.8 × 10^6^ ± 4.3 × 10^4c^
AAUMFM-6	86.67 ± 3.33^abcd^	4.51 (3.55–5.63)	2.1 × 10^6^ ± 1.9 × 10^5c^	90.00 ± 5.77^a^	4.80 (4.16–5.46)	2.7 × 10^6^ ± 2.5 × 10^5bc^

Mean with different letters in a column indicates the significant difference at Tukey’s HSD test, P < 0.05

Concerning lethal time at 50% (LT_50_) mortality values, isolates fell within the range of 3.22 to 9.26 days for *B. tabaci* and 3.08 to 8.16 days for *T. vaporariorum* nymphs (Table 2). *B. bassiana* AAUMFB-77 achieved the least LT_50_ values of 3.22 days and 3.08 days on *B. tabaci* and *T. vaporariorum* respectively. The spore production of isolates on whitefly nymph cadavers varied significantly among isolates (P < 0.001) with profuse sporulation ranging from 1.3 × 10^5^ to 6.5 × 10^6^ on *B.tabaci* and 1.6 × 10^5^ to 5.8 × 10^6^ conidia/cadaver on *T. vaporariorum*.

Regarding the multiple dosage (1 × 10^5^–1 × 10^8^ conidia/ml) response evaluations, the *B. bassiana* AAUMB-29 revealed the lowest LC_50_ values of 6.8 × 10^4^ conidia/ml on *T. vaporariorum*, (Additional file 1: Table S2) and 2.7 × 10^4^ conidia/ml on *B. tabaci* (Additional file 1: Table S3). Isolate *B. bassiana* AAUMFB-77 achieved the lowest LC_90_ value of 1.9 × 10^6^ conidia/ml against *B. tabaci*, whilst *B. bassiana* AAUMB-29 exhibited the lowest (1.5 × 10^6^ conidia/ml) against *T. vaporariorum.*

## Discussion

The main important bio-insecticidal traits of entomopathogenic fungi are the production of cuticle degrading extracellular enzymes [27]. Consequently, isolates of *B. bassiana,* and *M. anisopliae* were produced chitinase, lipase, and protease enzymes. The data showed that isolates differed in their chitinase, lipase, and protease enzyme activities that are attributed to their intraspecific and interspecific variability [28]. The three potential isolates, *B. bassiana* AAUMB-29*, B. bassiana* AAUMFB-77, and *M. anisopliae* AAUDM-43 displayed a high level of chitinase, lipase, and protease activities. Hence, the greater chitinase, lipase, and protease activities of isolates indicate the capability of protein, chitin, and lipids breakdown by these isolates. This alludes to that the fungal isolates are capable of successful penetration of insect cuticles [29, 30], with a high virulence effect against target insects [31].

In this study, the *M. anisopliae* AAUDM-43 induced the highest mortality of 94.27% on *B. tabaci* whereas *B. bassiana* AAUMFB-77 inflicted greater mortality of 95.37% on the *T. vaporariorum* adults after 10 days of treatment at the rate of 1 × 10^7^ conidia/ml. Among the isolates, *B. bassiana* AAUMB-29 displayed the highest mortality (98.33%) of nymphs on *B. tabaci* and both isolates of *B. bassiana* AAUMB-29 and *B. bassiana* AAUMFB-77 achieved 100% nymph mortality on *T. vaporariorum.* Several studies also confirmed the great bio-efficacy of *B. bassiana* and *M. anisopliae* against *B. tabaci* and *T. vaporariorum*, with mortality values of 86.47 to 96.61% at the concentration of 1 × 10^6^ conidia/ml in Italy [13], 71 to 86% at 1 × 10^7^ conidia/ml in Mexico [32]. The disparities in bio-control efficiency of isolates could be due to differences in conidial viability, spore concentrations, the vulnerability of pests, enzymatic activities, and experimental conditions [33, 34].

Spore production on the surface of target insect cadavers is one of the important parameters for the selection of candidate biological control agents. The *B. bassiana* AAUMFB-77 yielded the highest numbers of spores with 6.5 × 10^6^ conidia/cadaver on *B. tabaci* and 5.8 × 10^6^ conidia/cadaver on *T. vaporariorum* nymphs. These numbers were slightly higher than the number of spores produced; 7.9 × 10^5^ conidia/cadaver of whitefly nymph with *B. bassiana* [22], but lower than spore production by *B. bassiana* (8.3 × 10^7^ per beetle cadaver) [35]. The difference in spore production of fungal isolates on insect cadavers might be due to variation in humidity, fungal isolate, host species, experimental method, host stage, and body size [36]. With regard to the median lethal concentration, *B. bassiana* AAUMB-29 which was highly effective against the nymphs of whitefly species gave the lowest LC_50_ values of 6.8 × 10^4^ conidia/ml on *T. vaporariorum* (Additional file 1: Table S2) and 2.7 × 10^4^ conidia/ml on *B. tabaci* (Additional file 1: Table S3). The finding was slightly better than LC_50_ values on application with *M. anisopliae* and *B. bassiana* ranging from 0.22 × 10^4^ to 4.91 × 10^6^ conidia ml^−1^ against whitefly nymphs reported [37].

## Conclusion

This particular study showed that *B. bassiana* and *M. anisopliae* indicated differences in the production of chitinase, lipase, and protease enzymes. The *B. bassiana* AAUMB-29, *B.bassiana* AAUMFB-77, and *M.anisopliae* AAUDM-43 were the most virulent against whitefly nymphs and adults. The whitefly nymphs were more vulnerable to infection with *M. anisopliae* and *B. bassiana* than the adult stages of the whitefly species.

## Limitations

This study is limited to the enzymatic activities and bioassay study of *B. bassiana* and *M. anisopliae* against whiteflies under in-vitro conditions. A future study is required under field conditions to realize the efficiency of isolates for the development of myco-insecticide.

## Supplementary Information

**Additional file 1: Table S1.** Sources of* Beauveria bassiana *and* Metarhizium anisopliae *isolates used in this study. **Table S2.** The probit analysis of lethal concentrations values of *B. bassiana *and *M. anisopliae* in multiple dose-mortality response bioassays against *T. vaporariorum* nymphs 10 days post-fungal application. **Table S3.** The probit analysis of lethal concentrations values of *B. bassiana *and *M. anisopliae* in multiple dose-mortality response bioassays against *B. tabaci *nymphs 10 days post-fungal application. **Figure S1.** Extracellular activities of entomopathogenic fungi. Lipase activity of *B. bassiana *AAUMFB-77 (A), protease activity of *B. bassiana *AAUMB-29 (B), and Chitinase activity of *M. anisopliae *AAUDM-43 (C). **Figure S2.** Cultures of selected *M. anisopliae *and *B. bassiana *isolates on potato dextrose agar media. *B. bassiana *AAUMB-29 (A), *B. bassiana *AAUMFB-77 (B), and *M. anisopliae* AAUDM-43 (C). **Figure S3.** The mortality of whitefly adults with entomopathogenic fungi on tomato leaves. The mortality of whitefly adults by *B. bassiana *AAUMFB-77 (A), *B. bassiana *AAUMB-29 (B), and *M. anisopliae *AAUDM-43 (C).

## Data Availability

The datasets used and/or analyzed during the current study available from the corresponding author on reasonable request.

## Abbreviations

AREA: Average relative enzymatic activity
CL: Confidence limit
E: Excellent activity
EI: Enzymatic index
F: Fair activity
G: Good activity
EPF: Entomopathogenic fungi
LC_50_: The median lethal concentration required to kill 50%
P: Poor activity
PDA: Potato dextrose agar
REA: Relative enzymatic activity
SE: Standard error
