# Supplementary material for: Extracellular enzyme activity of entomopathogenic fungi, Beauveria bassiana and Metarhizium anisopliae and their pathogenicity potential as a bio-control agent against whitefly pests, Bemisia tabaci and Trialeurodes vaporariorum (Hemiptera: Aleyrodidae)
Source: BMC Res Notes. 2022 Mar 26;15:117. doi: 10.1186/s13104-022-06004-4 (PMC8961919; doi:10.1186/s13104-022-06004-4)
Supplement: Supplementary file 1 — Additional file 1: Table S1. Sources of Beauveria bassiana and Metarhizium anisopliae isolates used in this study. Table S2. The probit analysis of lethal concentrations values of B. bassiana and M. anisopliae in multiple dose-mortality response bioassays against T. vaporariorum nymphs 10 days post-fungal application. Table S3. The probit analysis of lethal concentrations values of B. bassiana and M. anisopliae in multiple dose-mortality response bioassays against B. tabaci nymphs 10 days post-fungal application. Figure S1. Extracellular activities of entomopathogenic fungi. Lipase activity of B. bassiana AAUMFB-77 (A), protease activity of B. bassiana AAUMB-29 (B), and Chitinase activity of M. anisopliae AAUDM-43 (C). Figure S2. Cultures of selected M. anisopliae and B. bassiana isolates on potato dextrose agar media. B. bassiana AAUMB-29 (A), B. bassiana AAUMFB-77 (B), and M. anisopliae AAUDM-43 (C). Figure S3. The mortality of whitefly adults with entomopathogenic fungi on tomato leaves. The mortality of whitefly adults by B. bassiana AAUMFB-77 (A), B. bassiana AAUMB-29 (B), and M. anisopliae AAUDM-43 (C). [file 13104_2022_6004_MOESM1_ESM.doc]

Table S1: Sources of*Beauveria bassiana*and*Metarhizium anisopliae* isolates used in this study

| Isolate code | Species | Accession  number | Collection sites |
| --- | --- | --- | --- |
| AAUMB-20 | *Beauveria bassiana* | MW077111 | East Shoa, Ethiopia |
| AAUMB-21 | *Beauveria bassiana* | MW077112 | East Shoa, Ethiopia |
| AAUMB-29 | *Beauveria bassiana* | MW077113 | East Shoa, Ethiopia |
| AAUKB-11 | *Beauveria bassiana* | MW077114 | East Shoa, Ethiopia |
| AAUMFB-77 | *Beauveria bassiana* | MW077115 | West Show, Ethiopia |
| AAUEB-59 | *Beauveria bassiana* | MW077116 | North Addis Ababa, Ethiopia |
| AAUMFB-5 | *Beauveria bassiana* | MW077117 | West Show, Ethiopia |
| AAUZM-18 | *Metarhizium anisopliae* | MW077118 | East Shoa, Ethiopia |
| AAUDM-43 | *Metarhizium anisopliae* | MW077119 | East Shoa, Ethiopia |
| AAUMFM-6 | *Metarhizium anisopliae* | MW077120 | West Show, Ethiopia |
| AAUEM-30 | *Metarhizium anisopliae* | MW077121 | North Addis Ababa, Ethiopia |
| AAUZM-60 | *Metarhizium anisopliae* | MW077122 | East Shoa, Ethiopia |

Table S2: The probit analysis of lethal concentrations values of *B. bassiana* and
 *M. anisopliae* in multiple dose-mortality response bioassays against *T. vaporariorum*
 nymphs 10 days post-fungal application

| Concentration  (conidia/ml) | % Mortality of *T. vaporariorum* (Mean ± SE) | | | | |
| --- | --- | --- | --- | --- | --- |
|  | AAUMB-21 | AAUMFB-77 | AAUMFM-6 | AAUMB-29 | AAUDM-43 |
| 1×10^5^ | 51.67±3.33^a^ | 66.67±1.67^a^ | 54.67±3.33^a^ | 61.66±4.40^a^ | 60.00±2.89^a^ |
| 1×10^6^ | 63.33±1.66^ab^ | 70.00±2.89^b^ | 58.00±4.40^a^ | 78.33±6.00^b^ | 71.67±3.33^b^ |
| 1×10^7^ | 85.00±4.40^a^ | 100.00±0.00^b^ | 90.00±5.7^ab^ | 100.00±0.00^b^ | 95.00±2.89^ab^ |
| 1×10^8^ | 88.33±2.88^a^ | 100.00±0.00^a^ | 91.67±6.00^a^ | 100.00±0.00^a^ | 100.00±0.00^a^ |
| Probit analysis summary | | | | |  |
| LC_50_  95% CL | 1.0 ×10^5^  4.3×10^4^ -5.7×10^5^ | 8.2 ×10^4^  4.6 ×10^4^-2.5×10^5^ | 8.3×10^4^  5.6×10^3^-2.42×10^5^ | 6.8×10^4^  3.0×10^3^-1.9×10^5^ | 7.2 ×10^4^  2.1×10^3^ -2.6×10^5^ |
| LC_90_  95% CL | 6.7 ×10^7^  9.8×10^7^ – 6.7×10^9^ | 2.6×10^6^  9.0×10^5^ -4.1×10^7^ | 5.6×10^7^  8.3×10^6^ -6.4×10^9^ | 1.5×10^6^  5.7×10^5^ -2.2×10^7^ | 4.5×10^6^  1.3×10^6^ -9.8×10^7^ |
| Slope ± SE | 0.42 ± 0.14 | 0.33 ± 0.15 | 0.31 ± 0.13 | 0.61 ± 0.19 | 0.54 ± 0.18 |
| Intercept± SE | 2.29±0.93 | 4.16±0.24 | 2.22±0.94 | 4.54±1.64 | 3.45±1.23 |
| X^2^ | 0.24 | 3.29 | 1.65 | 1.25 | 1.62 |
| *P-*value | 0.002 | 0.001 | 0.003 | 0.001 | 0.001 |

LC_50_ = the median lethal concentration required to kill 50%; LC_90_ = the median lethal concentration
 required to kill 90%; SE = standard error, CL=confidence limit

Table S3: The probit analysis of lethal concentrations values of *B. bassiana* and
 *M. anisopliae* in multiple dose-mortality response bioassays against *B. tabaci* nymphs 10
 days post-fungal application

| Concentration  (conidia/ml) | % Mortality of *B. tabaci* (Mean ± SE) | | | | |
| --- | --- | --- | --- | --- | --- |
|  | AAUMB-21 | AAUMFB-77 | AAUMFM-6 | AAUMB-29 | AAUDM-43 |
| 1×10^5^ | 55.00±2.89^ab^ | 66.67±4.40^b^ | 50.00±2.89^a^ | 65.00±2.88^ab^ | 56.66±4.40^ab^ |
| 1×10^6^ | 63.33±1.66^a^ | 73.33±1.67^ab^ | 80.00±10.40^b^ | 70.00±2.89^ab^ | 68.33±1.66^a^ |
| 1×10^7^ | 80.00±2.89^a^ | 98.33±2.86^b^ | 86.66±3.33^ab^ | 91.66±1.67^ab^ | 95.00±2.89^b^ |
| 1×10^8^ | 90.00±2.89^a^ | 100.00±0.00^b^ | 88.00±2.83^a^ | 100.00±0.00^b^ | 96.67±1.67^ab^ |
| Probit analysis summary | | | | |  |
| LC_50_  95% CL | 6.3 ×10^4^  2.7×10^3^ -4.9×10^5^ | 5.3×10^4^  9.2×10^3^-1.8×10^5^ | 7.5×10^4^  1.1 ×10^4^-3.3×10^5^ | 2.7×10^4^  3.6×10^3^-1.6×10^5^ | 5.4×10^4^  1.0×10^3^ – 3.1×10^5^ |
| LC_90_  95% CL | 1.3 ×10^8^  1.3×10^7^ – 3.2×10^10^ | 1.9×10^6^  6.4×10^5^ -3.8×10^7^ | 4.3×10^6^  2.9×10^5^ -1.1×10^7^ | 4.6×10^6^  1.9×10^6^ -1.0×10^7^ | 1.0×10^8^  2.6×10^7^ -4.6×10^8^ |
| Slope ± SE | 0.38 ± 0.14 | 0.45 ± 0.17 | 0.37 ± 0.13 | 0.50 ± 0.17 | 0.53 ± 0.16 |
| Intercept± SE | 1.85±0.92 | 3.84±1.47 | 2.01±0.95 | 2.55±1.17 | 2.84±1.05 |
| X^2^ | 0.11 | 2.63 | 1.12 | 0.76 | 1.10 |
| *P-*value | 0.008 | 0.002 | 0.004 | 0.003 | 0.001 |

LC_50_ = the median lethal concentration required to kill 50%; LC_90_ = the median lethal concentration
 required to kill 90%; SE = standard error, CL=confidence limit


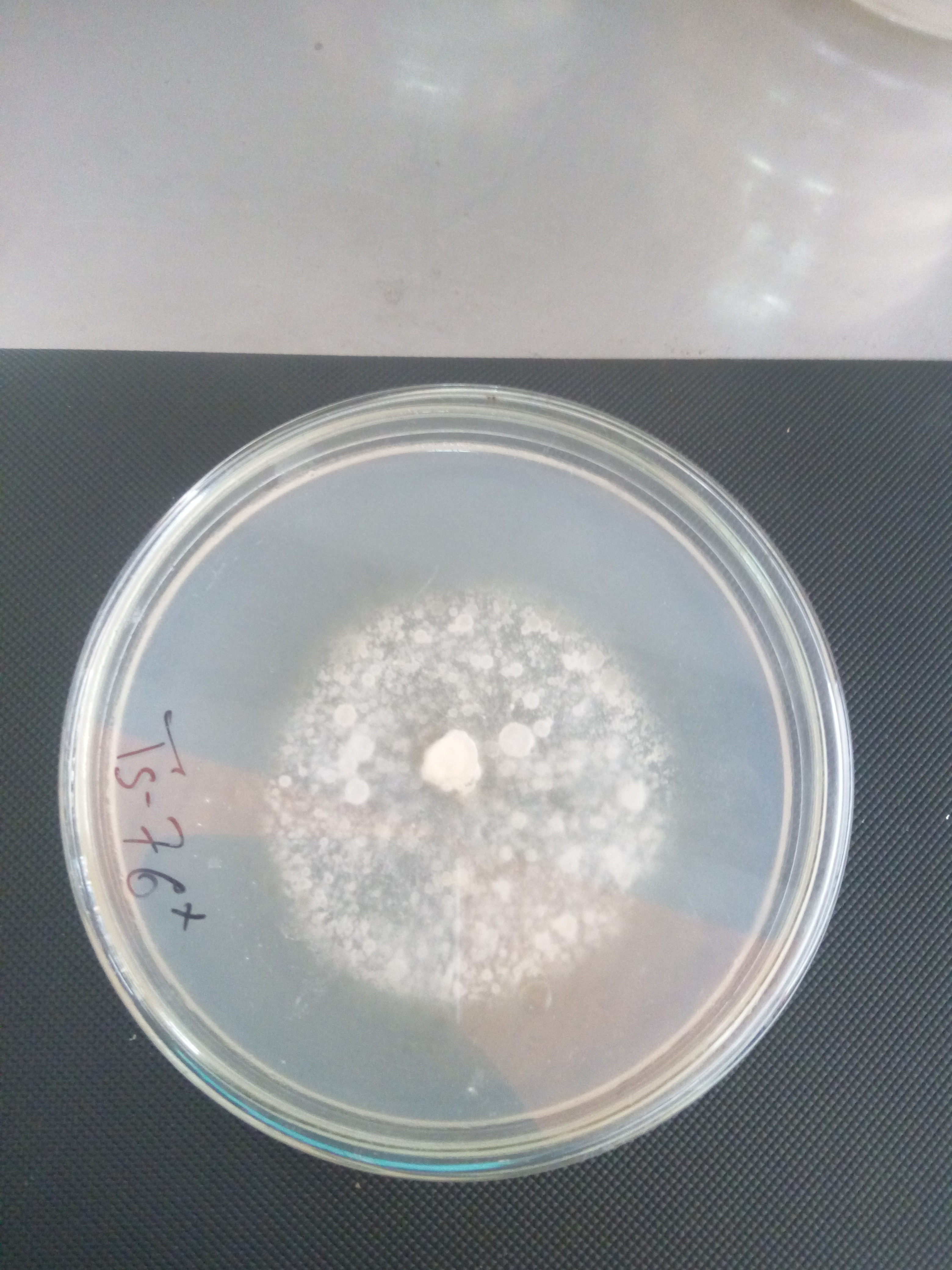

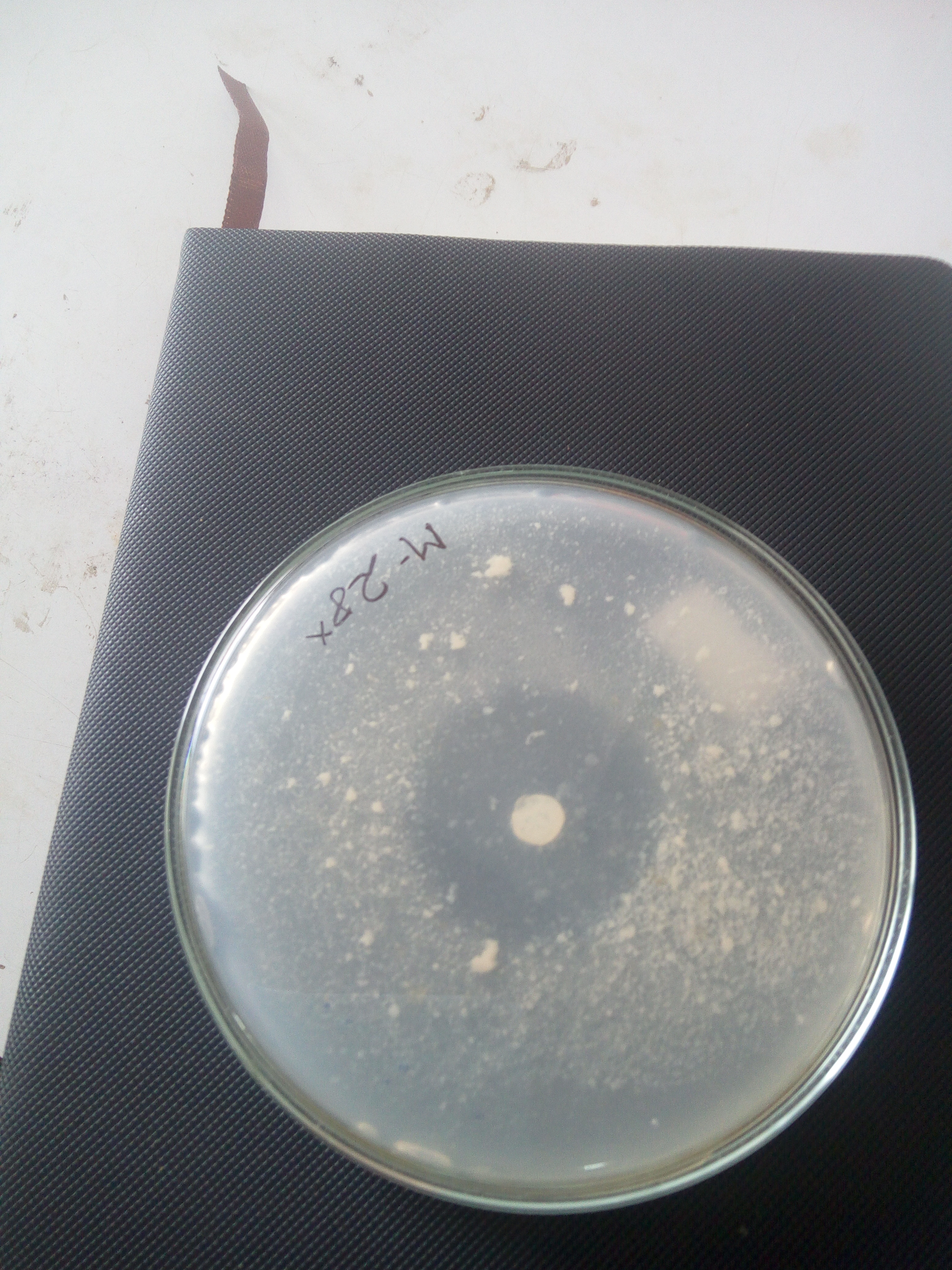

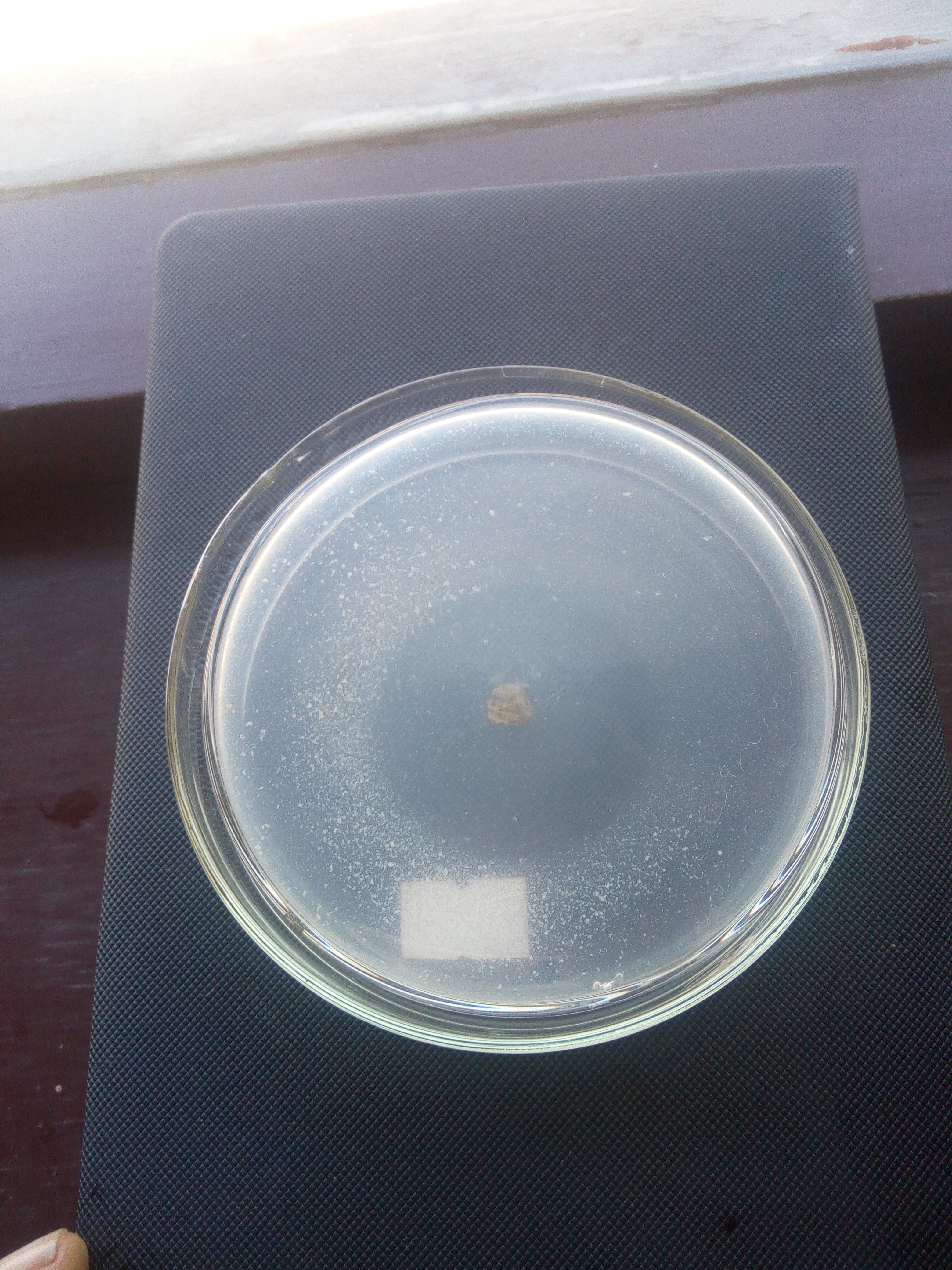


**A**

**B**

**C**

Figure S1: Extracellular activities of entomopathogenic fungi. Lipase activity of *B. bassiana* AAUMFB-77 (A), protease activity of *B. bassiana*AAUMB-29 (B), and Chitinase
 activity of *M. anisopliae* AAUDM-43 (C)


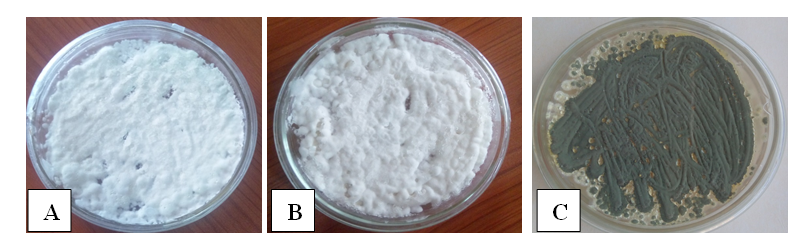


Figure S2: Cultures of selected *M. anisopliae* and *B. bassiana* isolates on potato dextrose agar
 media. *B. bassiana*AAUMB-29(A), *B.bassiana*AAUMFB-77(B), and *M. anisopliae*

AAUDM-43 (C).


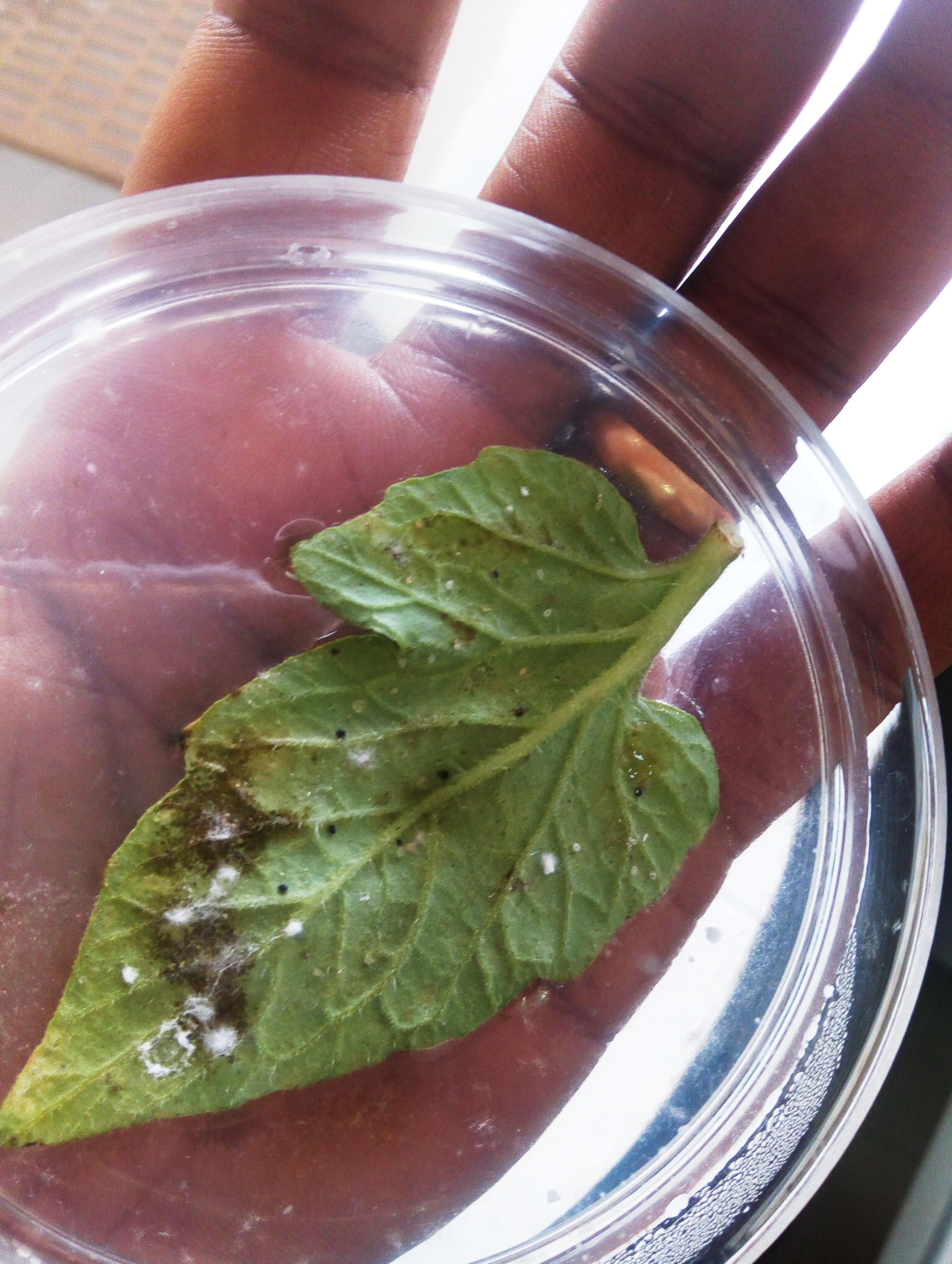

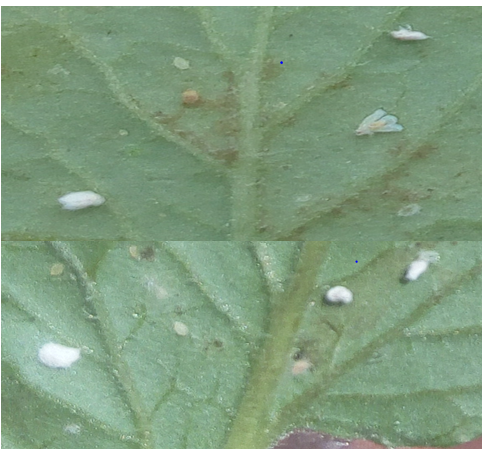

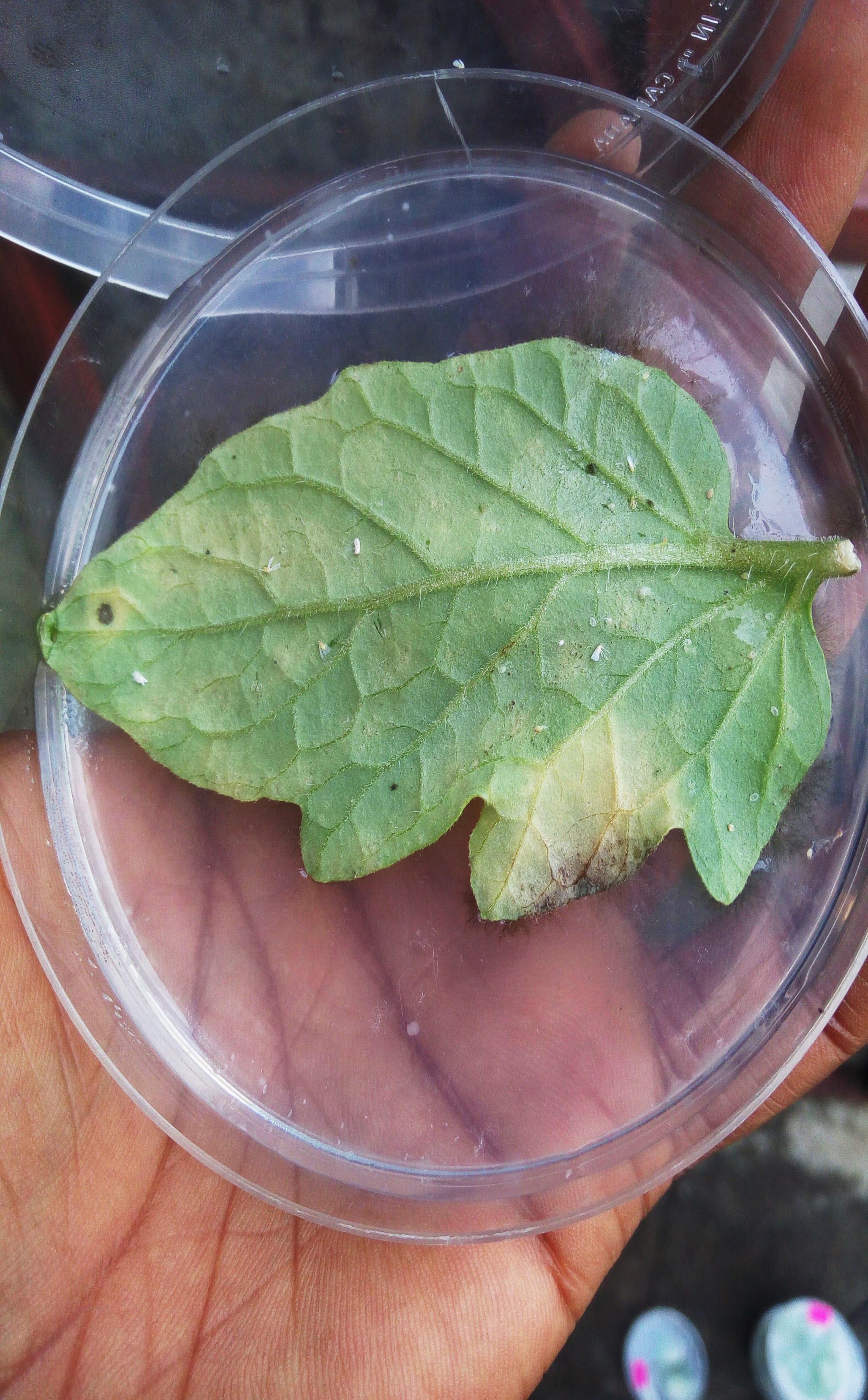


**A**

**B**

**C**

Figure S3: The mortality of whitefly adults with entomopathogenic fungi on tomato leaves. The
 mortality of whitefly adults by *B. bassiana* AAUMFB-77 (A), *B. bassiana* AAUMB-29
 (B), and *M. anisopliae*AAUDM-43 (C)
